# Becoming a nomad when hospitalized with a neurological disease: a phenomenological study

**DOI:** 10.1080/17482631.2020.1815487

**Published:** 2020-09-15

**Authors:** Malene Beck, Eileen Engelke, Regner Birkelund, Bente Martinsen

**Affiliations:** a Department of Neurology, Zealand University Hospital, Roskilde, Denmark; b Lienhard School of Nursing, Pace University, New York, NY, USA; c Teachers College, Columbia University, New York, NY, USA; d Section of Health Services Research Lillebaelt Hospital, University of Southern Denmark, Vejle Hospital, Vejle, Denmark; e Institute of Health, Department of Nursing Science, Aarhus University, Copenhagen, Denmark

**Keywords:** Hospital environment, sensory impression, aesthetics, placelessness, phenomenology, van Manen

## Abstract

Patients with a neurological disease are affected by their ability to maintain focus and are easily disturbed by outside stimuli. Few studies have investigated how sensory impressions from the physical environment contribute to patient’s wellbeing during hospitalization. However, no studies have explored the meaning of the environment to patients with a neurological disease during hospitalization. To understand what it is like to be a patient in a hospitalized environment at the neurological department. Nine patients were interviewed. Data analysis was inspired by the hermeneutic phenomenological methodology of van Manen. Four themes were identified: *Perceiving unrest leading to despair; Angling for attention from staff; Being in a vacuum of imposed passivity; Seeking breathing spaces*. The study provides insight into how environment plays a significant role in relation to existential issues for patients during hospitalization. Hence, the patients illuminate the experience of becoming nomads lurking around to find breathing spaces when they were not offered a calm and familiar environment. Patients shared that a hospital interior can be appealing and uplifting, decreasing their experiences of placelessness and thereby supporting them in a life situation where they feel less threatened concerning their health and wellbeing.

## Introduction

Patients suffering from neurological disease have one or more acquired or congenital conditions affecting the structure or function of the nervous or muscular systems (Digby & Bloomer, 2012; Feigin et al., 2016). Examples of neurological diagnoses include migraine, Parkinson’s disease, epilepsies, stroke, dementias such as Alzheimer’s disease, tumours or other subcategories of disease that negatively affect physical health, sensory perception and/or the cognitive capacity for planning, doing and decision making. Neurological conditions have a broad influence on human functioning at different levels. A characteristic of most neurological diseases is altered sensory experiences. For example, patients are often affected by a decreased ability to maintain focus and are easily disturbed by external stimuli (Digby & Bloomer, 2012).

The consequences of neurological disease are extensive and include the need for increased awareness of the importance of neurological patients’ surroundings and wishes (Beck, Poulsen et al. 2017a, 2017b; Beck et al., 2019). The importance of the environment for patients with dementia and individuals admitted to nursing homes has been described (Chaudhury et al., 2013), however, there is a lack of knowledge and understanding about how patients with neurological diseases experience the environment during their hospital stay (Day et al., 2000). The aim of this study was to explore the existential meaning of the hospital environment among people living with neurological diseases. Being able to understand the deeper meaning and significance of the hospital environment for neurological patients, will allow for interventions that improve the hospital experiences, and potentially their overall health, for these patients.

### Background

Knowledge about the importance of the environment in the treatment and care of illness is not new. In 1859, the founder of modern nursing, Florence Nightingale, described elements such as shape, colour, and light affecting physical healing. In recent years, researchers have focused on identifying features of the hospital environment that promote healing, wellbeing, and recovery of health (Lorenz, 2007).

Most research follows the paradigm of evidence-based design (Ulrich et al., 2004, 2008) Evidence-based design research has demonstrated the power of environmental design to support improved patient, family, and staff outcomes and to minimize or avoid harm in healthcare settings (Ullrich et al., 2014). Such studies have focused on the physical environment, exploring the effect of different environmental elements in the hospital on patients’ wellbeing (Høybye, 2013). However, although care has focused primarily on repairing physical issues, recognition is growing that the healthcare system could do more by promoting overall wellness, which requires expanding the focus to healing (Dubose et al., 2018).

Researchers highlight the importance of design in shaping hospital environments that provide a healing environment for patients (Høybye, 2013). Healing is understood in a wider sense that extends beyond environmental exposure and health outcomes; the environment can also support patients in discovering meaning during hospitalization (Egnew, 2005; Høybye, 2013). Hospital environments can be designed to become humanistic. Attending to environmental factors can significantly improve patients’ quality of life, as well as the experiences of hope and relief (Fontaine et al., 2001; Timmermann et al., 2015; Ullrich et al., 2014). Furthermore, the beneﬁcial effect of a supportive physical environment on patients’ health and wellness is clear, and research demonstrates remarkable results in terms of how physical surroundings positively inﬂuence their recovery and wellness (Ulrich et al., 2008).

Hospital environments that positively stimulate patients’ senses through colour, form, and interior design have been associated with uplifting effects on their mood and will to live (Martinsen, 2001, 2005, 2006). Researchers argue that the experience of wellbeing within the hospital environment is closely related to the degree the physical environment is homelike (Galvin & Todres, 2011; Todres & Galvin, 2010). In neurological care, the hospital environment is dominated by clinical sensory impressions, with less attention paid to aesthetics and homeliness (Beck et al., 2016, Beck et al., 2019). In this light, the hospital environment has significant potential for optimization, making it a better setting for patients to experience more supportive and comforting care. However, research into neurological care does not adequately address what is meaningful to patients when they are hospitalized with neurological diseases and how the environment can support their wellbeing during admissions.

## The study

### Aim

To understand what it is like to be a patient in a hospitalized environment at the department of neurology.

### Design

The study had a hermeneutic-phenomenological approach (van Manen, 2014) as we consider environmental aspects would impact significantly on the lived experiences of hospitalized patient afflicted by neurological disease. Thus, it was designed as a qualitative interview study (Kvale & Brinkmann, 2015) aiming at capturing patients’ experiences of the environment during hospitalization. Following the methodology of van Manen (2014), a phenomenological descriptive sensitivity is combined with an interpretive understanding of the lived experience and how it is given meaning (van Manen, 2014). The methodology is well suited to explore day-to-day practice and to reveal unknown or sensitive aspects of the depths and subtleties of other people’s experiences (ibid.); hence the interpretive approach gives voice to the human experience as it is (van Manen, 1997). In the phenomenological tradition, the concept of the lifeworld has been used to frame the world of everyday existence (van Manen, 2014). In coming to understand and explore the lifeworld, this study used the notion of lifeworld existentials to access and study the lifeworld of the neurology patients in the hospital environment. The existentials are; lived thing, lived body, lived time, lived space, lived other, lived technology, and is described as the fundamental structure of every persons’ lifeworld (van Manen, 1997, 2014).

### Participants

Nine patients were invited to participate in the study, including 3 women and 6 men. The women were aged 39–82 years and the men, 42–80 years. All patients suffered from a neurological diagnose e.g., epilepsy, Parkinson, migraine, multiple sclerosis, or brain tumour. Patients were admitted for treatment of symptoms of their condition, and not cure their disease. Some patients were permanently dependent on caregivers; the rest had varying needs for assistance with physical activities. All, but one of the patients’, had previous hospital admissions. Thus, it was expected that, the patients could provide nuanced insight on different places in the department given their diverse experiences of earlier admissions.

The patients’ day of admission was considered when information was collected, allowing us to study a variety of environmental experiences (Polit & Beck, 2010). The length of their stay ranged from a few days to several weeks. The number of patients were chosen to provide insight and a richly textured understanding of how patients assign meaning to the environment during hospitalization (Kvale & Brinkmann, 2015). Patients were selected on the basis of the likelihood that they would contribute information-rich data (Malterud, 2011) and on their varying admission dates, to allow for a greater range of patient experiences (Polit & Beck, 2010). This meant that the participants were able to verbally describe the hospital environment and participate in an interview that was conducted while walking or being wheeled around the hospital setting. Patients with severe cognitive deficits, expressive aphasia, and dementia were excluded, because their verbally ability to describe the phenomenon were limited. Patients were contacted in person by the first author (MB), who also informed them of the study aim.

### Settings

The department of neurology treats people with diseases of the brain, spinal cord, nerves, and muscles. The department has 60 beds and admits an average of four hundred patients a month. The department consists of two wards along two long corridors that consist of private rooms and two-bed rooms, in which light floor-to-ceiling curtains separate patients’ beds. Bedrooms are on one side of the ward and toilets and bathrooms are on the other side. The walls are white with large windows that admit natural daylight that stretches across the grey linoleum floors of patient rooms and into the hall. Room railings, cabinets, ceilings, and doors are painted in shades of purple. Wide dark green doors complement the purple hues. In the hallway, at the centre of the ward, white laminate tables and chairs are arranged in three groupings, creating spaces where patients and their families can sit. The chairs are made of wood and washable purple fabric. Wall sticker murals of green plants and posters with the message ‘Blood clot in the brain—CALL 112ʹ hang on the wall near the tables. At the end of the ward, extra tables, and exercise equipment, such as exercise bikes, a ball, and a treadmill, are set up. The hallway also holds various pieces of apparatus, screens, tables with vases, a refrigerator, and clean bedside tables.

### Views of the department

**Figure:**
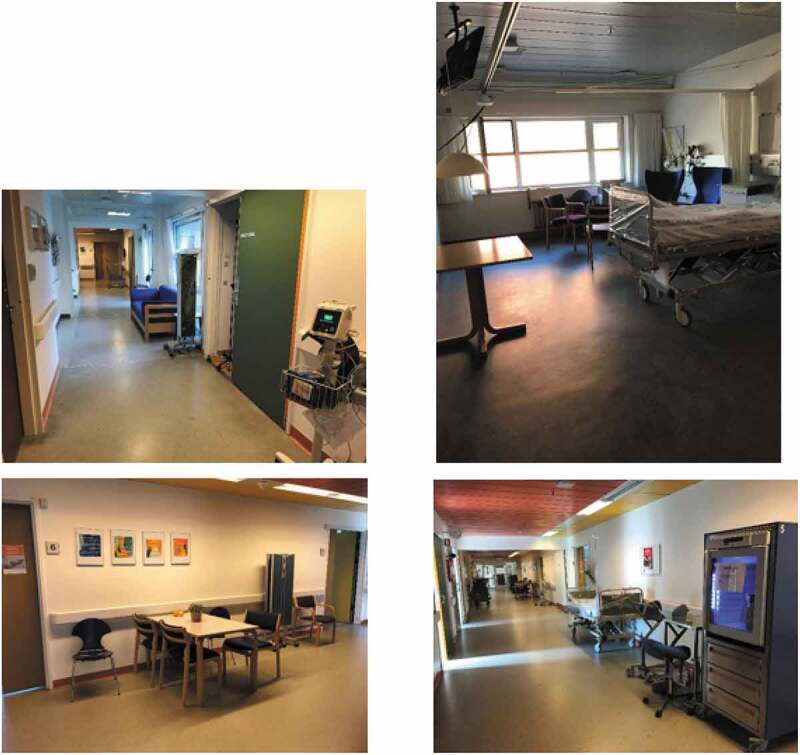

### Rigour

The basic strategy to ensure rigour in qualitative research is a systematic and self-conscious research design, data collection, interpretation and communication (Polit & Beck, 2010). Norlyk and Harder (2010), highlight that to make a phenomenological study, phenomenological, it is essential to clarify the principles which guided the research. Here, minimum scientific criteria must articulate methodology keywords, as well as describe how an open attitude was adopted.

The rigour in this study relies on the hermeneutic-phenomenological approach that systematically guided the whole research process, by investigating the phenomena of hospital environments. van Manen (2014) identifies “wonder” as an essential keyword within conducting phenomenological studies. In this study, our preunderstanding was found based on the phenomenological teachings that spaces and places are more than just physical and architectural settings. Spaces and places have existential meanings and are important attunements on how humans experience their being in the world (Martinsen, 2001; Norberg-Schulz, 1978; van Manen, 1997). Thus, as a premise in this study, a wondering of how the phenomena of hospital settings are meaningful to patients, was the overall guide. This meant that data was carefully collected until a substantial explanation and understanding of *what it is like* to be a patient in a neurological hospital environment, were obtained. Purposefully, to describe open-minded on how the phenomena of hospital environments can be interpreted and understood in future care to (neurological) patients.

### Ethical considerations

Written and verbal information about the study was given to all participants and informed consent was obtained. Participants were assured that their names and other personal information would be anonymized to maintain confidentiality. They were reassured that they could withdraw from the study at any time without any consequences for their treatment and care in the department. The study was approved by the Danish Data Protection Agency, which requires safeguarding of any personal information and securing the anonymity of participants (REG-081-2018). According to the Regional Ethical Committee, a qualitative interview study is not considered as biomedical research and requires no further approval. The study was performed in accordance with the ethical guidelines of the Nordic Nurses Federation and the Helsinki Declaration.

### Data collection

Individual semi-structured interviews took place from September 2018 to December 2018 in either a conference room or the patient’s bedroom. To capture the lived experience of patients and how it is to be a patient in the hospital environment, an interview guide, structured according to the six lifeworld existentials (van Manen, 2014), supported the interview. In practice, the interview guide was learned by heart, and it thereby came to function more like an invisible and implicit setting for the interview than an actual guide. The interviews started with an opening question: “*Please tell a little about a particular moment that you have been experiencing, while being hospitalized here?*”

According to van Manen (2014), it is essential to practice attentive attunement and open-mindedness while talking about lived experiences. Ideally, the interviewer would strive to put aside unwarranted assumptions while iteratively questioning and searching for the essence of the study phenomena (25). The interviewer did not have a daily presence at the department, and did not know the participants before the interviews. However, as a clinical researcher working within the field of general neurology, the interviewer (MB) knew that neurological patients could be sensitive to alterations in an environment. Thus, the phenomenological reduction required a continual reflexive effort to “lead back” to the question, “*Is this indeed what the experience of the environment may be like for a patient?*” (van Manen, 2014, p. 31). The exploration of lived experience was conducted in a manner that was critical and rigorous, yet also open and tentative (van Manen, 2017).

Openness in the interview situation, in the context of phenomenological research, is described as concerning not only listening to the spoken words in search of meaning, but also as possessing the ability to deeply absorb the interviewees’ expressions with both mind and body (Angel, 2013). Given the notion of the open-minded approach, awareness towards the importance of not taking over the conversation, but rather letting the patients tell their stories, and freely express the meanings they assigned to the phenomena of interest, was in focus. In practice this meant that the interviewer strived to allow the conversation to be driven by the patients, while also, continually returning back to the question of describing the experience. Knowing that people living with neurological diseases may need additional time to process and formulate their thoughts (Kirkevold & Bergdahl, 2007), each interview required patience, empathy and careful prompting to assure the patients felt comfortable and safe while describing their experiences. Secondary, probing questions such as, *“I really want to understand your situation, please take your time to go through what is important to you right here, right now*?”, were used to guide the discussion back to the phenomenon.

Allowing patients to tell their stories and freely express the meanings they assigned to their experiences is related to adopting a welcoming attitude (Kvale & Brinkmann, 2015). This attitude was achieved by creating a relaxed atmosphere using friendly and approachable body language (Fog, 2004). Senses and intuition were used in the interview to decide when to ask the patients to elaborate on their statements, to ask follow-up questions or to let silence and pauses take over (Angel, 2013).

Each interview was journaled (by MB) to capture the attunement of the encounter with the patient. Noting what the patient was wearing, how, where and with whom they were sitting, if they were near a window or if there was clutter in the bedroom provided an attunement of the interview moments; thus letting the phenomena of the hospital environment be disclosed. The interviews lasted for approximately 45 minutes and were recorded, reviewed, and transcribed verbatim. No patients declined participating in the interviews.

### Data analysis

The thematic analysis was conducted according to the analytic steps described by van Manen (2014), beginning with reading interview transcripts line by line, purposefully attending to embedded meanings. In this process MB & BM searched for descriptions to answer the question, “What is it like to be a patient hospitalized in the neurological department?”

First, interviews were read repeatedly with an open-minded attitude about the real-life experiences embedded in the overall sense of “what was going on” (van Manen 1997, 2006). Second, the text was broken into clusters of meaning. The clusters were analysed and interpreted in the context of the overall understanding of the interviews, by continuously going back and forth between clusters of meaning and the interview text as a whole. van Manen (2006) emphasizes that a phenomenological analysis is an enterprise of writing that aims at disclosing pre-reflective experiences. Pre-reflectiveness is understood as lived experiences as they occur and differs from arbitrary opinions (van Manen, 2014). Thus, the challenge was to look for and “bring to the surface from the depths of life’s ocean” (van Manen, 1997, p. 54) the quotations that illustrated the particular experience in a strongly evocative way (van Manen, 2017).

Finally, clusters were grouped into essential themes to capture the phenomenon of interest (van Manen, 1990). These were presented to co-authors (EE & RB) to validate the interpretation and arguments for clustering (van Manen, 2017). During the analysis, thematic statements were formulated as figures of meaning in concert with the above analytic reflective method to help point to possible eidetic meaning aspects of the phenomenon (van Manen, 1990). Eidetic refers to unique or more invariant patterns of meaning that may make a phenomenon distinct (van Manen, 2014). These thematic statements were used to structure the presentation of the text. According to van Manen (2014), existentials are intertwined and cannot always be presented as categorical as represented in theory. Our analysis presented the existentials that were suitable to answer the phenomenological question, of what it is like be a patient hospitalized in the neurological ward. The themes contain detailed descriptions of lived experiences with the goal of revealing the complexity and structure of meaning. Two authors (MB & BM) had extensive experience in neurological care, and all authors had experience with previous phenomenological studies.

Given the themes identified in the analysis phase, the perspectives of the Norwegian architect and philosopher Norberg-Schulz (1978, 1980, 1985) was included; purposefully to further understand the meaning of places to patients at the department of neurology in relation to their experience of existence during hospitalization. Norberg-Schulz (1978, 1980, 1985) was concerned about the meaning of places in relation to human existence and was inspired by Heidegger’s phenomenological theory of architecture. Norberg-Schulz (1978) emphasizes that places are existentially significant for humans, pointing out that attunement to a place can be experienced physically, as we recognize the rhythms and tensions of a place and become familiar with it.

## Findings

Four themes were identified from the data analysis: 1) perceiving unrest leading to despair; 2) angling for attention from staff; 3) being in a vacuum of imposed passivity; and 4) seeking breathing space. These themes are considered as figures of meaning that point to the many facets of lived meaning that may be related to the phenomenon of the environment for patients hospitalized in the neurological department.

### Perceiving unrest leading to despair

Patients expressed a sense of unrest as an unavoidable condition they had to deal with on their own. Unrest depended on others in the environment. For example, patients described how a constant, but underlying sound of people always talking, was annoying. The sensory impression from the environment was explained like an overload of impressions, in which the patients had difficulty sorting out which ones were important and which ones were not. This meant, that patients were often unnecessarily ready and experience being “on”, which over time, diminished their (environmental) demands and upset them, since a lack of quietness was needed to relax. The environmental sound was described as noise that the patients were forced to deal with but, that significantly negatively affected their existential experiences of being “present” in the department. Shaking his head and raising his voice, a young man answered rapidly to the question of how the sound contributed to the unrest:

There is a constant sound in here. It is that bipping sound. It really bothers me. When you lie there … and all the other sounds are added on. Cups and glasses clicking. Then it starts ringing in my ears (P1).

The constant noise was perceived as an overwhelming, innervating, negative stimulus from the environment that could not be suppressed. Patients could not decide what to hear, when to hear it, and how to get a break from noise.

Another source of unrest arose when elements of the environment were worn or destroyed. Carelessness about interior structures and/or furniture generated unrestful activity that contributed to noise loads and offended patients because it limited the possibility of experiencing silence and thus feeling calm. A worn-out environment had an existential meaning to these patients, because as the patients’ explained, it communicated a lack of care. The patients described that this lack of care of their own environment could possibly translate to a lack of care to them. The patients expressed, that having a broken door meant that care is not occurring in the environment, and they then questioned their own care and whether they were getting the best care. They expressed, “if my healthcare workers are not taking care of the ‘details in the rooms, are they not taking care of the ‘details in my care?” This was illustrated when a middle age woman, quite frustrated, explains how the use of a broken door turned into a reflection on how caring is provided:

The doors over there, if they are open, then they drive back and forth and they creak all the time (swearing while grimacing). I don’t understand why they don’t use a doorstop that can hold the door. You are so tired and then suddenly … [makes a loud noise] (P4).

Restfulness in the environment depended on the actions of others, especially staff. Often, when staff members created a chaotic environment by talking loudly (P4), running instead of walking (P6), or moving hectically (P5) this negatively influenced patients’ overall impressions of care and treatment. When asked to describe the hospital environment, a young woman who had not been admitted to the hospital before, starts crying and explains how she had turned around and looked out the window just to avoid further contact with other patients. She said:

New people come every day, including staff. They (staff) dashes around. You just lie down and turn your back. It seems so sad (pointing at the hallway). Therefore, it is nice that you can look out the window. That becomes very important (P6).

Few spaces provided patients with the restful experiences they wanted, and these places were often difficult to find. Many patients reported that they were not allowed to walk around or walk outside due to their diagnoses, so they stayed in their beds. Coupled with the unrest in the environment, staying in bed could lead to feelings of despair related to many uncontrollable sensory experiences that patients described as inevitable when they were in the department. This situation exhausted the patients and worsened their disease status. A man expanded the meaning with the environment by explaining he had difficulties *just being himself* (P1). *H*e could not find inner peace. The inner peace he speaks about is related to complete quietness and he describes how the lack of this made him feel:

You can get desperate while being in here. It is primarily the sound because there are many who speak at once. You are continuously thinking of quiet places where you can hide. You ask yourself, ‘Where can I just be myself?’ That is very difficult to find. Inner peace. And it is due to the external environment because something is happening constantly … 24/7 (P1).

Another elderly man, who was depending on staff members to mobilize himself in bed, elaborated on his despair about the constant noise in the department by using the word “metastases”, which could indicate the noise from the environment being cancerous. He said:

You should try to record one night here. It’s totally scary. There is such a lot of loud noise. It’s pretty awful. You just wish you could do this (flicks finger) and the sound was just dead. It would really do something for your wellbeing. If you lie there and have metastases in the brain, they go straight up in the cerebellum (P4).

### Angling for attention from staff

Staff members had a great influence on patients’ experiences of the environment in the neurological ward. Accommodating and friendly staff members made the chaotic environment easier to bear and generated calm feelings for patients. As one said, “That you feel welcome and that you can feel calm. They (staff) are SO nice to come sit and talk for a bit. That is peaceful to me” (P2). However, the opposite was also true if they experienced staff members as disengaged and tired of their jobs. One patient said, “Some of them really signal that they don’t want to be here but, at the same time, I hear them fooling around in the corridor. Why aren’t they so accommodating to us?” (P5)

Although participants were self-reliant, they continually angled for contact with staff and tried to find out who they could connect with. The angling was aligned with the journaled notes, where it was identified that patients were trying to make eye contact with the staff when, for example, we were walking in the hallway for coffee. Another instance, a staff member came into the room during an interview, and the patient smiled and asked a humorous question inviting them into the conversation It was evident at times, the difficulty for patients to figure out staff members’ identities and professions, because staff members sometimes did not introduce themselves or even address patients at all. As a young woman said, while looking down the table “Actually I feel left to myself while I am waiting for the scanning (…) I would prefer, that they [the staff] came over to me and said “hi” or something like that (…) Then I wouldn´t feel ignored (P5).

Overall, the attitude of staff members played a significant role in whether the environment was an uplifting experience for patients. When patients experienced contact with staff members as trustful and positive, the white walls and lack of art or other environmental artefacts became less important and hospitalization was easier to bear. Conversely, staff members in a bad mood or who did not engage, caused patients to withdraw to spaces outside the ward (e.g., the parking lot) where they expressed, it was better to be alone and find respite. Like other patients, a mother of three children admitted for observation of a possible tumour, was greatly worried, and explained the differences between moving from the inside environment to the outside environment. She described being on abench outside the department, where she illuminated on how breezes, colours and freedom to make her own caring choices, was possible to cultivate. She said:

If you socialize with people who are negative, you get negative (…) so sometimes it’s important to go down to the bench to watch the leaves blowing in the wind and the tree (…) it provides me some kind of rest (…) a change of scenery, because otherwise it’s very boring (P5).

### Being in a vacuum of imposed passivity

Patients shared that they were bored while hospitalized. Nobody expected them to do anything except be ready for examinations, treatment, or care when the staff eventually appeared. Nor did patients have anywhere to go to distract themselves. To pass time, they could only look out of the window, read a book, or drift around inside and outside the ward. A man, who needed time to formulate his valuable thoughts said quietly; “Nothing happens here. (…) Nothing (…). I’m waiting, making crosswords, reading, watching television and sleeping. That’s what you do (…) but if you just had some richly coloured paintings that you maybe could enjoy.” (P3). Thus, the patients did expand the understanding of the waiting time as a significant environmental aspect, by explaining how they experienced a lot of activity, but by virtue, the activity was rarely related to their individual care and treatment. It then became more “noise” than activity.

Patients had a strong wish for spaces in or outside the ward where they were not reminded of their disease, e.g., peaceful spaces where their thoughts could just wander off. Since the reality was quite different, it was up to the individual to find distractions. Some patients succeeded in finding spaces where they nearly forgot that they were admitted. For others, identifying such a relaxing space was nearly impossible:

I would really like to have a place where I could go and look at something [nice], to spend some time without thinking (…) I have preferred to have a bed near the window, so I could watch out of it. Now I’m just looking into the curtain [between the beds] (P5).

The environment in the neurological ward also included fellow patients. Having plenty of time on their hands meant that patients could closely watch how the staff treated and addressed other patients. Even if patients did not want to know anything about their fellow patients, shared rooms forced them to watch and overhear them. Listening to other patients’ private information or conversations was sensed with uneasiness, discomfort, and unfamiliarity of their own space. Patients could experience this as an indecent exposure that prompted them to leave their rooms and search for a space without burdensome sensory experiences. In addition, ill patients placed in the corridor could reduce mobile patients’ inclination to go out and wander there.

### Seeking breathing spaces

During the interviews, patients identified how small things or flowers from the garden, made them feel more at home. However, when asked to expand on any homelike moments or experiences while hospitalized, the patients noted that the environment in the neurological ward was dull and lacking any possibility of positive homelike distractions. Examples of environmental elements that eliminated the possibility of homeliness was; tables without chairs, grey walls, white boards with sloppy handwriting, the worn interior, and/or lamps hanging crookedly. A man, disabled to walk, starts laughing when asked to what is it like to be patient in is room; “They must have got this (pointing in the room) very cheap, since it looks like this.” (P4) Another man who was permanently dependent on caregivers was eager to describe how the uninspiring environment influenced him and suggested:

If they could just put some colorful pictures at the wall that you could be excited about and be looking at. Or maybe have something else to look out besides the clock. Well, having something that could get your thoughts to wander off (P7).

The dull environment and constant noise, were important factors that made patients describe a need for breathing spaces where they could be calm and uplifted by the surroundings, e.g., when looking at a tree on the parking lot. However, finding a space to “breathe” was difficult at the hospital. Patients who were mobile skulked around the building to find spaces to be themselves. One patient tells carefully, while smiling:

I also know a little about the basement down there (laughs), because I have been sitting there and then opened one of the doors to outside. It’s almost like a refuge. I sat there a lot while I was in treatment. There I sat down in the basement and cooled down (P1).

Other examples of breathing spaces for patients were the parking lot or the benches outside. Patients’ wishes for breathing spaces were simple. They longed for spaces they could go when they felt uncomfortable, where they could be alone and avoid noise and clinical activity. The patients explained that even though, they (in some cases) were in dependable of staff members and could leave the department for a walk, they often did not know where to go. The patients’ descriptions of what it is like to be in that situation illuminated a need for a hospitalized “hearth”; one that could provide an experience of homeliness, belonging, and calmness. This was significant, because searching for breathing spaces made patients restless and facilitated an overall experience of becoming a nomad within the confines of the hospital. A first time admitted young woman, starts crying when asked to describe what it is like to be a patient in a neurological hospital environment. She explains what provokes her decision to leave the department during the day:

Sometimes I walk out to the parking lot just to get out. When you have been here for a long time, you can’t help feeling really sick. You can feel trapped into the negative environment. It is about a particular atmosphere being present. Then it can be helpful to just sit at the bench outside, watch the wind or the trees to get that peaceful feeling. A change of scene, or else it will be too trivially (P5).

As illustrated in this quote, patients moved around restlessly to avoid unpleasant experiences and search for quiet spaces. Finding a peaceful place to rest was up to patients, who picked diverse and individual spaces.

## Discussion

An overall understanding of what it is like to be a patient in a hospital environment at the department of neurology highlighted that potentials in the hospital environment are untapped, but nevertheless play a significant role in relation to existential issues that patients are experiencing during hospitalization.

Our study showed that overall, patients experience a negative environmental existence within the confines of the hospital ward. This was emphasized consistently with an uneasiness from unwanted and uncontrollable noise. Patients with neurological diseases found it crucial to have quiet spaces to feel physically grounded. Aaron et al. (1996) and Fontaine et al. (2001) found that noise is an environmental hazard creating discomfort and disruptive sleep. In addition, based on the fact that excess noise increases cortisone and stress levels, the WHO developed guidelines for noise exposure in public outdoor places (Jarosinska et al., 2018). Nevertheless, insufficient attention has been paid to the effect of noise on patients’ existential condition during hospitalization. In our study, the patient experiences show that in a clinical context, a focus on a healthy or welcoming sound environment is lacking. This is consistent with the findings of Edvardsson et al. (2005), who interviewed patients and showed that exceeding their expectations for the environment was a mediating factor in sensing an atmosphere of ease. This insight highlights how the core of the patient’s environmental experiences is based on simple requests regarding positive experiences that can be supportive existentially. Sensing an atmosphere of ease facilitates patients’ ability to ground themselves in familiar surroundings; hence follow their own rhythms, their experiences of acknowledgement and feeling cared about.

Our study expands the understanding of experiencing unfamiliarity within the hospital environment, because the patients illuminated on a sense of not belonging to something meaningful during hospitalization. This corroborates research highlighting place as holding complex and unique meanings and that the experience of healthcare cannot be detached from the place in which it is received (Casey, 2001; Creswell, 2009). The sense of a place, with its colours, textures, and objects, constitute a kind of belonging that provides a degree of security, comfort, familiarity, continuity, and unreflective ease (Todres & Galvin, 2010). Our study provides new insight to the understanding of how a hospital environment is meaningful to people living with a neurological disease, by showing how closely related the physical environment is to patients’ homelike experiences. Based on the patients’ experiences we argue that simple physical homelike artefacts and basic care and repair of equipment, can stimulate patients’ senses and be an uplifting experiences that effects patients’ mood positively. This fosters comforting support in a troubled situation of living life with a neurological disease.

Furthermore, the study illuminates that the environment is significant for patients both as a bodily experience such as lying in a bed, and as a possibility of “being”, such as being allowed to enjoy stillness. Therefore, being familiar with the hospital environment and knowing how to be and where to be, can be an existential compass within the hospital walls. The Norwegian nurse and philosopher Kari Martinsen (2018) points out that familiarity with the hospital environment has the potential for facilitating homelike spaces in which reflexivity of what is important during sickness is enhanced. Our findings showed that the experience of feeling at home within the environment of the hospital was difficult for patients to achieve, despite constant attempts to search for it. Patients moved around, striving for healing spaces where they could be themselves and fulfill their needs for quietness and homeliness.

In addition, our study elucidates the fact that the existing environment caused patients to wander off, searching breathing spaces in which they could find existential footholds. This raises important questions about the significance of the placelessness that patients experienced during hospitalized. Placelessness can be described as having lost one’s foothold in existence (Norberg-Schulz, 1978). Norberg-Schulz (1978) highlight the importance of making the environment, such as a room, an existentially significant place and notes that places can bring humans back the elemental joys of life. Thus, nurturing patients’ sensory experiences means establishing a stable environment with positive distractions that connect people to the place the hospital represents. Research has shown that places like the hospital environment can connect people to each other as humans and hold the potential to ensure an experience of being taken care of during illness (Ullrich et al., 2014). This is consistent with our study findings that focusing on the significance of the hospital environment has the potential to provide patients with rest and security, despite the many constant changes they experience related to neurological disease. The environment can provide tired, vulnerable, and sick patients with the opportunity to be nourished and nurtured. However, to prevent them from becoming restless nomads, purposeful attention must be paid to how the environment can address patients’ needs for breathing spaces and alleviate existential challenges during illness.

### Limitations

Given our qualitative approach, the findings must be systematically evaluated to determine whether our conclusions can be generalized to other patients or settings (Kvale & Brinkmann, 2015). The definition of “analytic generalization” (Brinkmann, 2013) ensures that findings from one situation can be compared into another. This is a hermeneutic phenomenological study and to our knowledge the first study to explore the understanding of the hospital environment based on the experiences of people living with neurological diseases. Doing a hermeneutic phenomenological study has given us the unique opportunity to expand the understanding of how it is to be a patient afflicted with a neurological disease during hospitalization at the department of neurology.

It can be argued that a limitation in this study is the small amount of participants, in which representability is difficult to maintain. Due to the phenomenological–hermeneutic character of this study, quantity was not the priority (Polit & Beck, 2010). Therefore, the findings are considered contextual (Polit & Beck, 2010; van Manen, 2014), which means that other interpretations are likely to be found in other contexts and settings.

A limit may be that patients were all of the same cultural background, although having the strength of great variation in age, several admissions as well as length of admissions days. The findings in this study are specific to patients admitted to a danish neurological ward, and the significance of the environment to bedbound and/or immobilized patients and those in other settings should be investigated. These investigations could reveal new aspects of the phenomenon of environment, adding further knowledge about patients’ perspectives (Malterud, 2011).

## Conclusion

Our study showed that, if patients do not feel attached to a calm, aesthetically pleasant and familiar environment during hospitalizations, they become nomads, skulking around to find breathing spaces. Put simply, patients want an environment that is peaceful and quiet. Environmental characteristics are meaningful to patients in an existential way and reveal the interplay between the environment and patients’ experiences of unrest and discomfort during hospitalizations. Our study concludes that staff members’ engagement with patients in the environment and the possibility of experiencing quiet can be appealing and uplifting to patients and decrease their experiences of placelessness. Thus, they are supported in a situation in which they feel that their health and wellbeing are both threatened.

### Relevance to clinical practice

The relevance of the study lies in its potential to inform hospital managers and staff members about the importance of the environment to patients hospitalized with neurological diseases. A calm and inspiring environment must be designed systematically; our study highlights the fact that a supportive environment does not happen by accident. Deeper insight is also needed into how patients assign meaning to interventions focusing on creating welcoming and comforting environments.
